# Rapidly Progressive Frontotemporal Dementia With Amyotrophic Lateral Sclerosis in an Elderly Female

**DOI:** 10.7759/cureus.32182

**Published:** 2022-12-04

**Authors:** Yazeed G Sweedan, Muhammad Haroon Khilan, Rahul Rane, Ashish Jain, Saba Waseem

**Affiliations:** 1 Internal Medicine, Conemaugh Memorial Medical Center, Johnstown, USA

**Keywords:** wernicke encephalopathy, dementia, variant creutzfeldt-jakob disease, familial amyotrophic lateral sclerosis, frontotemporal syndrome

## Abstract

A 69-year-old female with a family history significant for early onset dementia and a past medical history significant for coronary artery disease, primary hypertension, type two diabetes mellitus, and Crohn’s disease presents to our facility with rapidly progressive cognitive decline, delusions, hallucinations, and ambulatory dysfunction over the past two months. Neurological examination was remarkable for bilateral horizontal nystagmus, tongue fasciculations, bilateral upper extremity incoordination, and bilateral lower extremity spasticity, atrophy, and weakness. Laboratory and microbiological testing were remarkable for low serum thiamine levels. Computed tomography (CT) of the head without contrast showed significant brain atrophy in the frontal and temporal regions as compared to a CT without contrast of the head 5 years prior. Magnetic resonance imaging (MRI) of the head with and without contrast showed significant atrophy in the frontal and temporal regions as well as the cerebellum. Follow-up electromyography was consistent with lower motor neuron disease. The patient was given adequate thiamine supplementation for her thiamine deficiency and discharged on donepezil with instructions to follow up with the amyotrophic lateral sclerosis clinic for further monitoring and initiation of riluzole.

## Introduction

Frontotemporal dementia (FTD) is a clinically and pathologically heterogeneous disorder characterized by disturbances in behavior, personality, and language accompanied by focal degeneration of the frontal and/or temporal lobes [1]. It is one of the most common causes of early onset dementia, with a mean age of onset of 58 years old [1,2]. Studies suggest that up to 50% of patients with FTD have at least one relative with dementia, and an autosomal-dominant inheritance pattern is observed in up to 25% of patients [3]. The most common disease-causing genetic mutations include those in microtubule-associated protein tau (MAPT), the granular precursor (GRN) gene, and a noncoding hexanucleotide expansion in chromosome nine open reading frame 72 (C9orf72) [4]. Clinically, it can be sub-classified into behavioral variant FTD, semantic variant primary progressive aphasia, and non-fluent variant primary progressive aphasia [2]. The behavioral variant is the most common clinical subtype, named after its hallmark presentation of behavioral and personality changes early in the disease course [2].

## Case presentation

A 69-year-old female with a family history significant for early onset dementia and a past medical history significant for coronary artery disease, primary hypertension, type two diabetes mellitus, and Crohn’s disease presents to our facility with rapidly progressive cognitive decline over the past two months. The cognitive decline was associated with delusions, hallucinations, ambulatory dysfunction, and a complete lack of insight.

Neurological examination was remarkable for bilateral horizontal nystagmus, tongue fasciculations, bilateral upper extremity incoordination, and bilateral lower extremity spasticity, atrophy, and weakness (muscle strength of 2/5). Patellar reflexes were 1+ bilaterally while the rest of the reflexes were absent except for plantar reflexes which were downgoing bilaterally. Finger to nose test was representative of dysmetria. On cognitive testing, the patient demonstrated disorientation, tangential conversation, and was unable to complete the serial sevens test or count from 10 to one backward. Bedside swallow evaluation demonstrated impaired swallowing.

Initial laboratory testing including complete blood count, complete metabolic panel, C-reactive protein, lactic acid, troponin, magnesium, and phosphorus was unremarkable. Urine analysis was positive for bacteriuria. B12 was within normal limits but folic acid was low. Further testing showed low Vitamin B1. Antinuclear antibodies (ANA) and paraneoplastic antibody panels were negative. Urine cultures grew Enterococcus faecalis, but the rest of the microbiological testing including blood cultures, hepatitis panel, and Lyme disease antibody test was negative.

**Figure 1:**
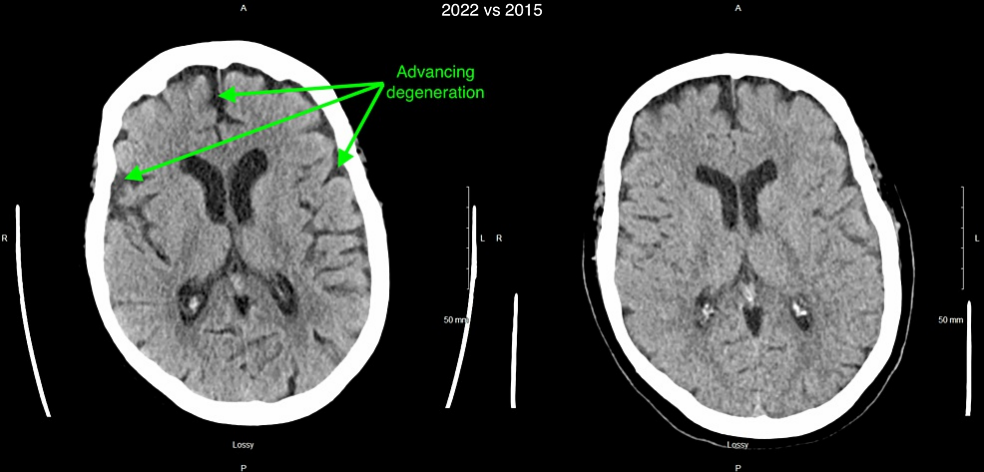
Computed tomography (CT) without contrast of the head 2022 vs 2015 (1) 2022 scan is on the left while 2015 scan is on the right

**Figure 2:**
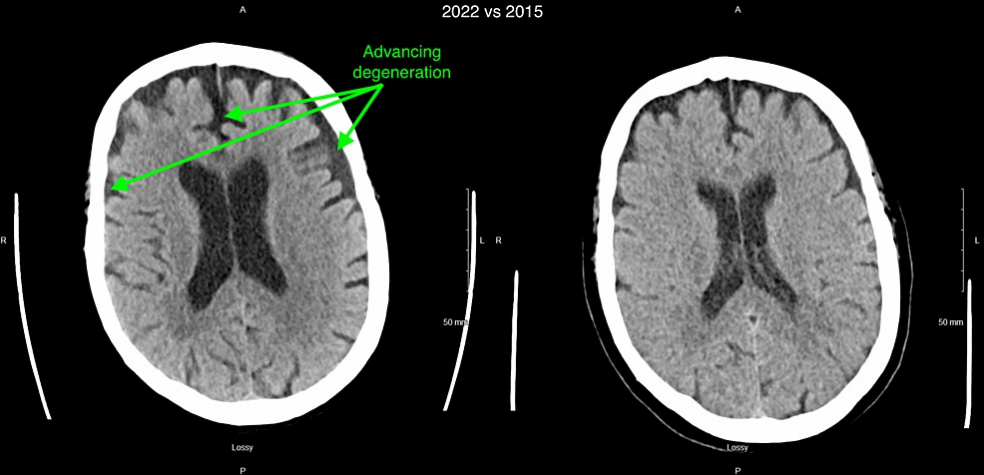
Computed tomography (CT) without contrast of the head 2022 vs 2015 (2) 2022 scan is on the left while 2015 scan is on the right

**Figure 3:**
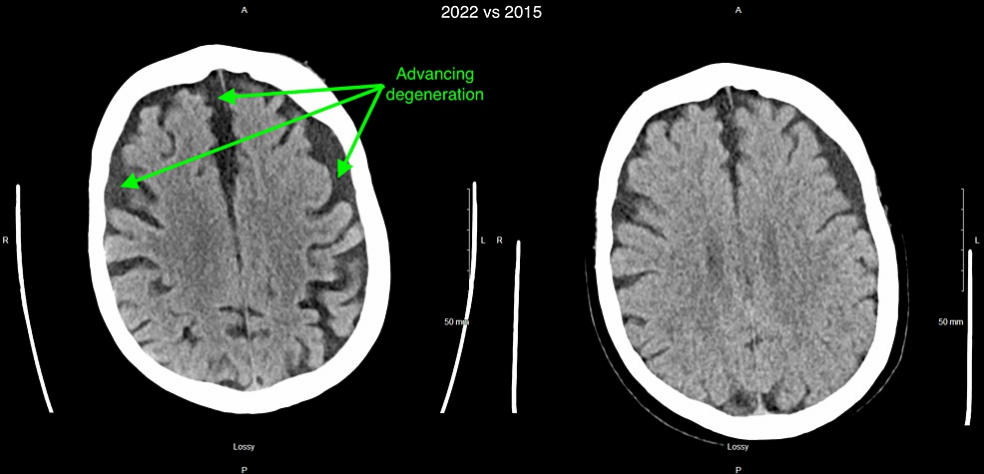
Computed tomography (CT) without contrast of the head 2022 vs 2015 (3) 2022 scan is on the left while 2015 scan is on the right

**Figure 4:**
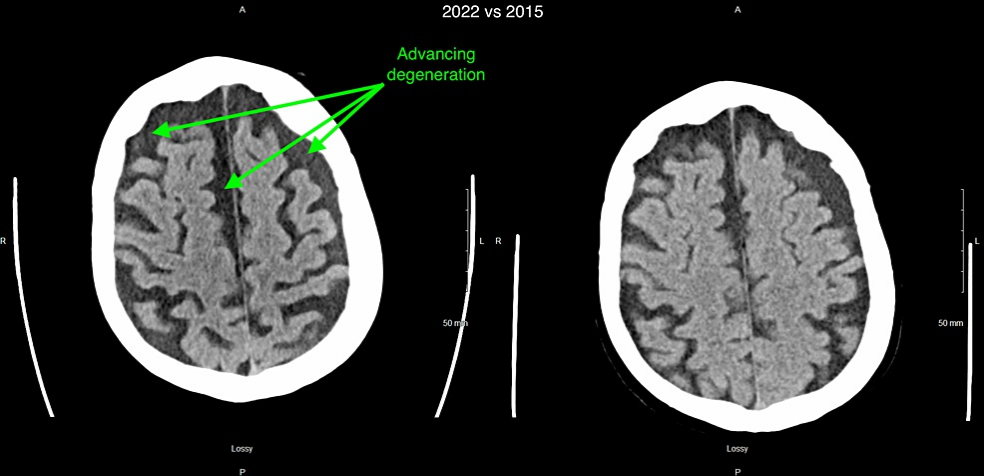
Computed tomography (CT) without contrast of the head 2022 vs 2015 (4) 2022 scan is on the left while 2015 scan is on the right

**Figure 5:**
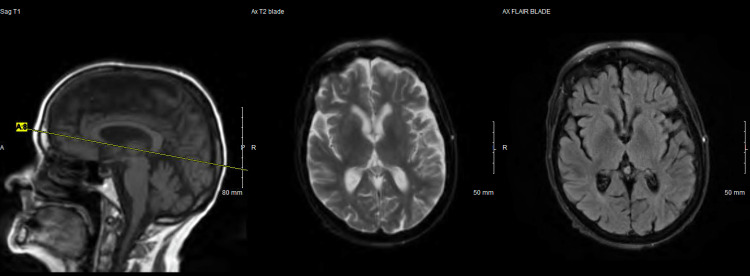
Magnetic resonance imaging with/without contrast of the head 2022 (1)

**Figure 6:**
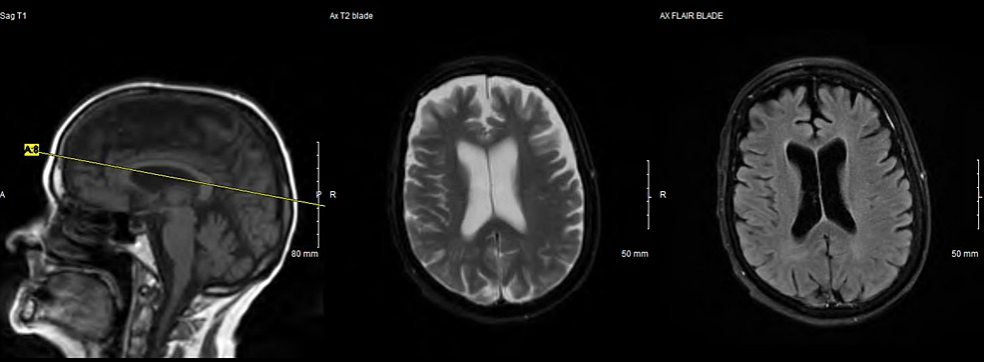
Magnetic resonance imaging with/without contrast of the head 2022 (2)

**Figure 7:**
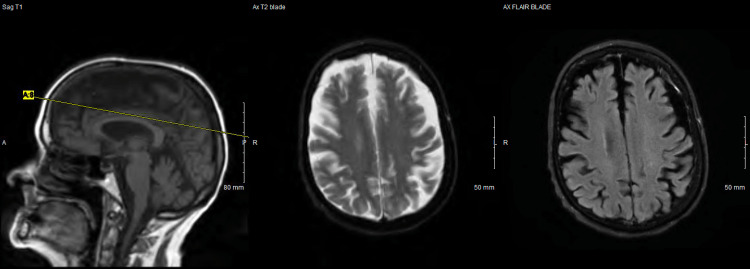
Magnetic resonance imaging with/without contrast of the head 2022 (3)

Computed tomography (CT) scan of the head without contrast was remarkable for significant brain atrophy in the frontal and temporal regions as compared to a CT head more than 5 years prior (Figures 1-4). Magnetic resonance imaging (MRI) of the head with and without contrast showed significant atrophy in the frontal and temporal regions as well as the cerebellum (Figures 5-7). Lumbar puncture drained out the clear, colorless fluid. Cerebrospinal fluid analysis including cell count, gram stain, aerobic culture, and anaerobic culture was unremarkable. Glucose and protein levels were within normal limits and there were no 14-3-3 proteins. Panels for meningitis, encephalitis, and multiple sclerosis were unremarkable. Electromyography was consistent with lower motor neuron disease.

The patient was given adequate thiamine supplementation for her thiamine deficiency and ampicillin for her urinary tract infection. Before discharge, the patient was started on donepezil by neurology and instructed to follow up with the amyotrophic lateral sclerosis clinic for further monitoring and initiation of riluzole. The patient’s condition continued to rapidly deteriorate leading her to return to the hospital one month later for comfort care and passing away the next day.

## Discussion

FTD is a clinically and pathologically heterogeneous disorder characterized by disturbances in behavior, personality, and language accompanied by focal degeneration of the frontal and/or temporal lobes. It is one of the most common causes of early onset dementia, with a mean age of onset of 58 years old [1,2]. Studies suggest that up to 50% of patients with FTD have at least one relative with dementia, and an autosomal-dominant inheritance pattern is observed in up to 25% of patients [3]. The most common disease-causing genetic mutations include those in MAPT, the granular precursor (GRN) gene, and a noncoding hexanucleotide expansion in chromosome nine open reading frame 72 (C9orf72) [4].

Clinically, it can be sub-classified into behavioral variant frontotemporal dementia (bvFTD), semantic variant primary progressive aphasia, and non-fluent variant primary progressive aphasia [2]. The behavioral variant is the most common clinical subtype, named after its hallmark presentation of behavioral and personality changes early in the disease course [2]. Early behavioral changes include disinhibition, apathy, loss of sympathy/empathy, hyper-orality, preservative/compulsive behaviors, and dysexecutive neuropsychological profile, with most patients lacking insight into their behavioral changes [2].

Approximately 20% of patients with the behavioral variant develop concomitant motor neuron disease, particularly amyotrophic lateral sclerosis (ALS) [5]. C9orf72 repeat expansion is the most common genetic cause of familial bvFTD and ALS [6]. ALS due to this expansion is associated with earlier disease onset, bulbar disease, higher frequency of co-morbid FTD, and a shorter age-matched median survival as compared to sporadic ALS [7]. Carriers of this expansion are also more likely to exhibit psychotic symptoms including delusions and hallucinations as compared to other FTD patients [8].

There are currently no effective disease-modifying treatments for FTD. Both pharmacologic and non-pharmacologic interventions are aimed at ameliorating symptoms, particularly the behavioral symptoms of FTD. Survival from the onset of symptoms in patients with bvFTD with ALS is approximately 2 years [9].

Sporadic Creutzfeldt-Jakob disease (sCJD) is a rapidly progressive prion disease with highly variable clinical and neuropathological findings that eventually leads to death in less than a year. The most common age of presentation is around 64 years old. The clinical characteristics that are most diagnostic are progressive mental deterioration and myoclonus [10]. The most sensitive marker for sCJD is a positive CSF 14-3-3 [11]. Hyperintense signal on diffusion-weighted imaging DWI, fluid-attenuated inversion recovery (FLAIR), and T2-weighted images involving the cerebral cortex and corpus striatum caudate head and putamen is the most common pattern on MRI in patients with sporadic CJD [12]. Due to the highly variable presentation of sCJD and the similarities it shares with bvFTD and ALS, it was quite challenging to rule it out clinically. However, frontotemporal atrophy on MRI along with lack of CSF 14-3-3 made bvFTD with ALS more likely.

Wernicke encephalopathy (WE) is the best-known neurological complication of thiamine deficiency that usually presents with the classic triad of encephalopathy, oculomotor dysfunction, and gait ataxia [13]. Encephalopathy is characterized by profound disorientation, indifference, and inattentiveness [13]. The level of thiamine can be tested in the serum, however, the sensitivity and specificity of this blood test in symptomatic patients are unclear, as blood level may not accurately reflect brain thiamine level [13]. Typical MRI findings in WE include areas of increased T2 and FLAIR signals, decreased T1 signal, and diffusion abnormality surrounding the aqueduct and third ventricle and within the medial thalamus, dorsal medulla, tectal plate, and mammillary bodies [14]. Prompt administration of thiamine leads to improvement in ocular signs within hours to days [13]. Even though this patient had low serum thiamine levels, frontotemporal atrophy on MRI along with the lack of improvement after adequate thiamine supplementation made bvFTD with ALS more likely.

## Conclusions

FTD is a clinically and pathologically heterogeneous disorder characterized by disturbances in behavior, personality, and language accompanied by focal degeneration of the frontal and/or temporal lobes. Its most common clinical variant, the behavioral variant, can coexist with ALS usually when C9orf72 repeat expansion is present. MRI of the head to detect frontotemporal atrophy and electromyography to detect motor neuron disease are of vital importance when evaluating for bvFTD with ALS. They are also necessary to rule out other possible differentials that require different treatments.
